# Persistent endometrial dysfunction after fertility-sparing treatment for endometrial carcinoma: insights from donor oocyte cycles

**DOI:** 10.3389/fonc.2026.1897240

**Published:** 2026-07-24

**Authors:** Pierfrancesco Greco, Ermanno Greco, Maria Teresa Varricchio, Manuel Maria Ianieri, Valeria Masciullo, Flavia Costanzi

**Affiliations:** 1 Centre for Reproductive Medicine, Villa Mafalda, Rome, Italy; 2 Unit of Gynecology and Obstetrics, Policlinico Abano Terme, Abano Terme, Italy; 3 Division of Gynecological Surgery, Department of Women’s and Children’s Health, Fondazione Policlinico Universitario A. Gemelli Istituto di Ricovero e Cura a Carattere Scientifico (IRCCS), Rome, Italy; 4 Surgical and Medical Sciences and Translational Medicine, Sant’Andrea Hospital, Sapienza University of Rome, Rome, Italy

**Keywords:** assisted reproductive technology, endometrial carcinoma, endometrial receptivity, fertility-sparing treatment, oocyte donation

## Abstract

**Background:**

Fertility-sparing treatment (FST) is an accepted option for selected women with endometrial carcinoma (EC) or atypical endometrial hyperplasia (AEH) wishing to preserve fertility. However, the impact of FST on subsequent endometrial function remains poorly understood. Oocyte donation (OD) offers a unique model to evaluate endometrial receptivity independently of oocyte quality. We assessed reproductive outcomes after OD in women aged ≥40 years with a history of FST for EC or AEH.

**Methods:**

This retrospective case series included women aged ≥40 years with histologically confirmed Endometrioid endometrial carcinoma (EEC) or AEH who achieved complete response after FST and subsequently underwent blastocyst transfer through OD. All cycles involved vitrified-warmed donor oocytes, intracytoplasmic sperm injection, blastocyst culture, and elective single embryo transfer. The primary outcome was live birth rate (LBR) per embryo transfer.

**Results:**

Sixteen patients underwent 27 embryo transfer cycles. Median age at first transfer was 45.5 years. Embryological outcomes were excellent, with a 96.8% oocyte survival rate and 80.4% top-quality blastocysts. Despite adequate endometrial preparation in all cases, reproductive outcomes were poor. The cumulative LBR per embryo transfer was 18.5% (5/27), while the cumulative LBR per patient was 31.2% (5/16). Patients with disease recurrence showed lower live birth rates than those without recurrence. Endometrial thickness was not associated with implantation outcomes.

**Conclusion:**

Women aged ≥40 years undergoing OD after FST for EC or AEH experience reduced reproductive success despite optimal embryo quality. These findings are consistent with the hypothesis of persistent endometrial dysfunction after FST and highlight the need for improved assessment of endometrial receptivity and timely integration of assisted reproductive treatment following complete response.

## 1 Introduction

Endometrial carcinoma (EC) is the most common gynaecological malignancy in high-income countries, with approximately 417,000 new cases and 97,000 deaths reported annually worldwide (1). Atypical endometrial hyperplasia (AEH), the recognized precursor lesion of EC, shares similar hormonal and metabolic risk factors, including obesity, chronic anovulation, polycystic ovary syndrome, and prolonged unopposed oestrogen exposure (2). Although EC predominantly affects postmenopausal women, an increasing number of diagnoses are being made in women of reproductive age who have not yet completed childbearing. Approximately 2–5% of EC cases occur in women younger than 40 years, most of whom are nulliparous (3). In addition, EC or AEH may be incidentally identified during infertility work-up in up to 3% of patients undergoing assisted reproductive evaluation (4).

Standard treatment for EC consists of total hysterectomy with bilateral salpingo-oophorectomy, resulting in definitive loss of fertility (5). However, in carefully selected patients with AEH or early-stage, well-differentiated EC confined to the endometrium, in particular the endometrioid histotype, hereinafter referred to as endometrioid endometrial carcinoma (EEC), fertility-sparing treatment (FST) with progestins represents an accepted alternative for women wishing to preserve reproductive potential (6, 7). Current FST strategies commonly include levonorgestrel-releasing intrauterine system (LNG-IUS), alone or combined with systemic progestins such as megestrol acetate (8). Although complete histological response rates are relatively high, in young women with well-differentiated stage IA EEC undergoing fertility-sparing conservative management, the most recent meta-analyses estimate a pregnancy probability of approximately 25–60% and a live birth rate of approximately 20–70%, depending on the progestogen regimen employed and the duration of follow-up (9, 10).

Women aged 40 years and older undergoing FST represent a particularly challenging subgroup. Advanced reproductive age is frequently associated with diminished ovarian reserve, often limiting the feasibility and effectiveness of IVF with autologous oocytes and making oocyte donation (OD) the most realistic reproductive option (11–13). At the same time, both the disease itself and prolonged progestin exposure may adversely affect endometrial receptivity. Recent studies demonstrate that endometrial aging involves molecular, cellular, and epigenetic alterations, including impaired stromal decidualization, disrupted glandular architecture, and downregulation of implantation-related biomarkers, which collectively reduce implantation potential (14, 15). Moreover, repeated hysteroscopies and endometrial biopsies required during oncological surveillance can contribute to chronic inflammation, intrauterine adhesions, and further endometrial damage, exacerbating the decline in uterine receptivity with age (16).

In this setting, OD provides a unique biological model to evaluate endometrial function independently from the confounding effects of ovarian aging and oocyte quality. By using oocytes derived from young donors, implantation and pregnancy outcomes may more directly reflect the functional integrity of the endometrial environment after FST.

Despite the increasing clinical relevance of this population, evidence regarding reproductive outcomes after FST remains limited and largely derived from younger women undergoing IVF with autologous oocytes (17–19). Data specifically addressing OD outcomes in women aged ≥40 years with a history of EEC or AEH are currently lacking.

The aim of the present retrospective case series was therefore to describe reproductive outcomes following OD blastocyst transfer in women aged 40 years or older who underwent FST for EEC or AEH, with particular focus on implantation and live birth outcomes in this clinically complex population.

## 2 Materials and methods

### 2.1 Study design

We conducted a retrospective case series at the Reproductive Medicine Centre of Villa Mafalda Clinic, including women with histologically confirmed EEC or AEH who underwent FST followed by embryo transfer through OD.

The study was conducted in accordance with the principles of the Declaration of Helsinki and was approved by the Institutional Ethics Committee of Casa di Cura Villa Mafalda (Protocol No. CE 03/2026). All patients had provided written informed consent for clinical treatment and assisted reproductive procedures prior to enrolment.

### 2.2 Patient selection

Patients were eligible for inclusion if they fulfilled all the following criteria. A histologically confirmed diagnosis of EC, including endometrioid endometrial carcinoma (EEC), FIGO Grade 1 or 2, or AEH, established by curettage or hysteroscopic biopsy, was required. All patients must have completed FST with documented complete histological response prior to assisted reproductive technologies (ART) initiation and must have received oncological clearance to proceed with ART following two consecutive negative histological evaluations: the first after 3 months of progestin-based therapy and the second hysteroscopy after 3 months with directed endometrial biopsy. Additionally, patients were required to be aged 40 years or older at the time of the first embryo transfer and to have undergone at least one blastocyst transfer using donated oocytes performed at our institution following FST.

Patients were excluded if they had undergone embryo transfer before EEC/AEH diagnosis or before completion of FST or had undergone intrauterine insemination rather than embryo transfer. Further exclusion criteria comprised the presence of untreated uterine pathology known to independently impair implantation, including submucous leiomyomas or unresected endometrial polyps, as well as incomplete clinical records.

### 2.3 Fertility-sparing treatment and oncological follow-up

FST is considered for patients with endometrioid endometrial carcinoma classified according to the FIGO 2023 (20) staging system as Grade 1 (G1) or Grade 2 (G2), Stage IA, without myometrial invasion and without additional risk factors. Given the limited evidence supporting FST in Grade 2 (G2) EEC, its application in this subgroup should be evaluated on an individual, case-by-case basis.

Accurate histopathological characterisation is a prerequisite for FST eligibility. Hysteroscopy-guided endometrial biopsy is preferred over blind biopsy techniques for confirming the diagnosis. Conservative management may additionally be considered in women with early-stage EEC G2 (Stage IA) or with well-differentiated EEC G1 exhibiting minimal myometrial invasion (1–2 mm) (21, 22).

FST was administered in accordance with contemporary oncological guidelines throughout the study period. Two therapeutic approaches were employed: orally administered megestrol acetate (160–320 mg/day) or medroxyprogesterone acetate (400–600 mg/day) combined with an LNG-IUS, or LNG-IUS monotherapy alone. Endometrial surveillance was conducted at regular intervals throughout treatment. The first histological assessment was performed after three months of therapy, and in patients showing no evidence of residual neoplastic or pre-neoplastic disease, a second confirmatory evaluation was subsequently carried out via hysteroscopy with directed endometrial biopsy after a further three-month interval. Complete response was defined as the absence of EEC or atypical endometrial hyperplasia (AEH) on both consecutive assessments. Eligibility to proceed with assisted reproductive technology (ART) was granted exclusively following confirmation of complete response and formal oncological clearance. Disease recurrence was defined as the histological reappearance of EEC or AEH after a previously documented complete response; patients in this situation underwent additional FST cycles and repeat histological surveillance prior to reconsideration for ART.

### 2.4 Oocyte donation cycles and embryo transfer protocol

All OD cycles used vitrified-warmed oocytes obtained from anonymous donors younger than 34 years who had undergone controlled ovarian stimulation and oocyte retrieval at our centre. Fertilization was performed exclusively by intracytoplasmic sperm injection (ICSI). For all procedures, semen samples used for ICSI met normozoospermic criteria according to the World Health Organization (WHO) reference values (23).

Embryos were cultured to the blastocyst stage (Day 5 or 6) in a continuous single culture medium (Global Total, LifeGlobal, Guilford, CT, USA) under standardized conditions (37 °C, 6% CO_2_, 5% O_2_). Blastocysts were graded according to the Gardner and Schoolcraft morphological classification (Gardner and Schoolcraft, 1999), and all transfers were elective single blastocyst transfers (eSET).

Endometrial preparation followed a standardized hormone replacement therapy protocol. Pituitary down-regulation was achieved with a GnRH agonist (triptorelin, Decapeptyl; Ipsen Pharmaceuticals, Paris, France) prior to estrogen priming. Oral estradiol valerate (Progynova; Bayer AG, Leverkusen, Germany) was then administered with progressive dose escalation from 2 to 6 mg/day from cycle day 2 onwards until endometrial thickness reached ≥7 mm with a trilaminar pattern on transvaginal ultrasound. Luteal phase support was initiated with intramuscular progesterone (Prontogest; IBSA, Lugano, Switzerland) (50 mg/day), and blastocyst transfer was scheduled on the sixth day of progesterone exposure. Transfers were performed only when endometrial thickness on the day of progesterone initiation was ≥7 mm.

All embryo transfers were carried out under transabdominal ultrasound guidance using a soft transfer catheter (Wallace; Smiths Medical, Dublin, Ireland). Blastocyst selection was based on morphological grade according to Gardner and Schoolcraft (24).

### 2.5 Outcomes and statistical analysis

Clinical and laboratory data were extracted from electronic medical records, including age, BMI, histological diagnosis, FST regimen, recurrence status, embryological parameters, endometrial thickness at transfer, and reproductive outcomes.

The primary outcome was live birth rate (LBR) per embryo transfer cycle, defined as the number of live births divided by the total number of transfer cycles performed. Live birth was defined as delivery of a live infant beyond 24 weeks of gestation (25).

Secondary outcomes included positive β-hCG rate, clinical pregnancy rate, biochemical pregnancy rate, spontaneous abortion rate, and cumulative live birth rate per patient.

Continuous variables were reported as mean ± standard deviation or median with interquartile range, according to data distribution. Categorical variables were expressed as frequencies and percentages. Given the descriptive nature of this retrospective case series and the limited sample size, analyses were primarily descriptive. Exploratory subgroup comparisons according to recurrence status, histological diagnosis, and FST regimen were performed using Fisher’s exact test. Statistical analyses were conducted using IBM SPSS Statistics, version 28.0 (IBM Corp., Armonk, NY, USA).

## 3 Results

### 3.1 Patient characteristics

Sixteen patients met the inclusion criteria and were included in the analysis. **Table 1** summarizes baseline demographics and clinical characteristics, and **Table 2** presents individual patient data. The median age at first blastocyst transfer was 45.5 years (IQR 44–48; range 42–49). All 16 patients were nulliparous at the time of study enrolment. Male partner karyotype was normal (46, XY) in all 16 couples.

**Table 1:** Baseline demographic and clinical characteristics of the study cohort.

Variable	Total (n=16)	EC G1 (n=9)	EC G2 (n=3)	AEH (n=4)
Age at diagnosis, years, median (IQR)	44 (42–47)	43 (42–45)	42 (42–43)	46 (42–49)
Age at first ET, years, median (IQR)	45.5 (44–48)	45 (43–47)	43 (43–45)	47 (43–49)
Age range, years	42–49	42–49	42–45	42–49
BMI, kg/m², median (IQR)	27.6 (23.5–28.7)	27.9 (21.5–29.1)	27.8 (21.5–29.1)	27.1 (21.6–28.6)
Nulliparous, n (%)	16 (100%)	9 (100%)	3 (100%)	4 (100%)
FST: Megestrol acetate + LNG-IUS, n (%)	11 (68.8%)	7 (77.8%)	2 (66.7%)	2 (50.0%)
FST: LNG-IUS alone, n (%)	5 (31.2%)	2 (22.2%)	1 (33.3%)	2 (50.0%)
Disease recurrence, n (%)	9 (56.2%)	5 (55.6%)	2 (66.7%)	2 (50.0%)
Total ET cycles per patient, median (IQR)	2 (1–2)	2 (1–2)	1 (1–2)	2 (1–2)
Endometrial thickness, 1st ET, mm, median (IQR)	9.8 (8.5–10.7)	9.8 (8.5–10.8)	9.6 (8.6–10.5)	9.7 (7.5–10.9)
Time from FST completion to first ET, months, median (IQR)	10.5 (8.0–12.1)	10.4 (8.1–12.3)	9.8 (8.5–11.0)	11.0 (8.3–12.1)
Time from diagnosis to first ET, weeks, median (IQR)	41.5 (38.8–44.8)	/	/	/

EC, endometrial carcinoma; AEH, atypical endometrial hyperplasia; FIGO, International Federation of Gynecology and Obstetrics; BMI, body mass index; OD, oocyte donation; DOR, diminished ovarian reserve; FST, fertility-sparing treatment; LNG-IUS, levonorgestrel intrauterine system; ET, embryo transfer; IQR, interquartile range; SD, standard deviation.

**Table 2:** Individual patient characteristics and reproductive outcomes.

Pt	Diagnosis	Age (yr)	BMI	FST	Recur- rence	ET cycles	1st ET beta+	1st ET LB	2nd ET beta+	2nd ET LB
1	EEC G1	45	29.1	Meg+LNG-IUS	Yes	1	Yes	No	—	—
2	AEH	49	27.3	LNG-IUS	Yes	2	No	No	Yes	No
3	AEH	46	28.6	Meg+LNG-IUS	No	2	No	No	No	No
4	EEC G1	49	29.0	Meg+LNG-IUS	Yes	2	No	No	No	No
5	EEC G2	42	21.5	Meg+LNG-IUS	Yes	2	No	No	No	No
6	EEC G1	43	21.6	Meg+LNG-IUS	No	1	Yes	**Yes**	—	—
7	EEC G1	45	24.1	LNG-IUS	Yes	2	No	No	No	No
8	EEC G1	47	28.0	Meg+LNG-IUS	No	1	Yes	**Yes**	—	—
9	EEC G2	45	29.1	Meg+LNG-IUS	No	2	No	No	No	No
10	EEC G1	49	27.3	LNG-IUS	Yes	2	No	No	Yes	Yes
11	AEH	46	28.6	LNG-IUS	No	2	No	No	No	No
12	EEC G1	49	29.0	Meg+LNG-IUS	No	2	No	No	No	No
13	EEC G2	42	21.5	Meg+LNG-IUS	Yes	2	No	No	No	No
14	EEC G1	43	21.6	Meg+LNG-IUS	No	1	Yes	**Yes**	—	—
15	EEC G1	45	24.1	Meg+LNG-IUS	Yes	2	No	No	No	No
16	AEH	47	28.0	LNG-IUS	Yes	1	Yes	**Yes**	—	—

Pt, patient; EEC, Endometrioid endometrial carcinoma; G1/G2, FIGO Grade 1/Grade 2; AEH, atypical endometrial hyperplasia; Meg+LNG-IUS, megestrol acetate plus levonorgestrel-releasing intrauterine system; DOR, diminished ovarian reserve; ET, embryo transfer; LB, live birth; —, not applicable (single ET cycle). Bold values denote live birth achieved.

Histological diagnoses were as follows: EEC Grade 1, Stage IA without myometrial invasion and without risk factors in 9 patients (56.3%), EEC G2 stage IA without myometrial invasion and without risk factors in 3 patients (18.8%), and AEH in 4 patients (25.0%). The primary indication for OD was advanced maternal age.

Regarding FST: 11 patients (68.8%) received combined megestrol acetate (160 mg/day) plus LNG-IUS, and 5 patients (31.2%) received LNG-IUS monotherapy. The median time from FST completion to the first embryo transfer was 10.5 months (IQR 8.0–12.1). The median time from initial diagnosis to first embryo transfer was 41.5 weeks (IQR 38.8–44.8), corresponding to approximately 9.6 months (IQR 8.9–10.3). Patients with disease recurrence had a longer diagnostic-to-ET interval compared to those without recurrence (44.0 weeks, IQR 41.2–47.2 vs. 40.0 weeks, IQR 36.2–41.5), reflecting the additional time required for retreatment and repeat histological surveillance prior to ART re-initiation. Disease recurrence after an initial complete response occurred in 9 of 16 patients (56.2%), requiring additional courses of FST before proceeding with ART. Of the 16 patients, 5 underwent a single ET cycle and 11 underwent two ET cycles (total: 27 cycles).

### 3.2 Embryological parameters

**Table 3** summarizes embryological data for the cohort. The mean number of oocytes thawed per donor batch was 7.8 ± 0.7, with an oocyte survival rate of 96.8% (7.5 ± 0.7 oocytes surviving per batch). The median number of blastocysts obtained per cycle was 6 (IQR 5–6). Of 92 total blastocysts assessed, 74 (80.4%) were classified as top-quality (Grade A), 6 (6.5%) as Grade B, and 12 (13.1%) as Grade C. The median number of blastocysts cryopreserved per patient was 3 (IQR 2–3). The median endometrial thickness at the time of the first transfer was 9.8 mm (IQR 8.5–10.7; mean ± SD: 9.7 ± 1.5 mm), with no statistically significant difference between the first and second transfer cycles (9.8 mm vs. 10.1 mm; p = 0.67). All 27 transfers were single blastocyst transfers (100% SET compliance).

**Table 3:** Embryological parameters of the study cohort.

Parameter	Value
Total oocytes thawed, mean ± SD	7.8 ± 0.7
Oocyte survival rate, %	96.8%
Surviving oocytes, mean ± SD	7.5 ± 0.7
Total blastocysts obtained per patient, median (IQR)	6 (5–6)
Top-quality blastocysts (Grade A), n (%)	74/92 (80.4%)
Grade B blastocysts, n (%)	6/92 (6.5%)
Grade C blastocysts, n (%)	12/92 (13.1%)
Blastocysts cryopreserved per patient, median (IQR)	3 (2–3)
Endometrial thickness at 1st ET, mm, median (IQR)	9.8 (8.5–10.7)
Endometrial thickness at 2nd ET, mm, median (IQR)	10.1 (8.7–10.4)
Single embryo transfer (SET), n/total ET (%)	27/27 (100%)

HRT, hormone replacement therapy; GnRH-a, gonadotropin-releasing hormone agonist; SET, single embryo transfer; SD, standard deviation; IQR, interquartile range.

### 3.3 Reproductive outcomes

**Table 4** displays reproductive outcomes by transfer cycle and cumulatively. At the first transfer cycle (n = 16), five patients (31.3%) achieved a positive β-hCG, four (25.0%) developed a clinical pregnancy, one experienced a biochemical pregnancy (6.3%), one had a spontaneous abortion (6.3%), and four achieved a live birth (LBR = 25.0%). At the second transfer cycle (n = 11), two patients (18.2%) had a positive β-hCG, one reached clinical pregnancy (9.1%), one experienced a biochemical pregnancy (9.1%), one had a spontaneous abortion (9.1%), and one achieved a live birth (LBR = 9.1%). Across all 27 transfer cycles, the cumulative outcomes per ET were: positive β-hCG 25.9% (7/27), clinical pregnancy rate 18.5% (5/27), biochemical pregnancy 7.4% (2/27), spontaneous abortion 7.4% (2/27), and live birth rate 18.5% (5/27). The cumulative LBR per patient was 31.2% (5/16). No ectopic pregnancies were recorded.

**Table 4:** Reproductive outcomes per transfer cycle and cumulative per patient.

Outcome	1st ET (n=16)	2nd ET (n=11)	Overall (n=27 ET)	Cumulative LBR per patient (n=16)
Positive β-hCG, n (%)	5 (31.3%)	2 (18.2%)	7 (25.9%)	—
Biochemical pregnancy, n (%)	1 (6.3%)	1 (9.1%)	2 (7.4%)	—
Clinical pregnancy rate, n (%)	4 (25.0%)	1 (9.1%)	5 (18.5%)	—
Spontaneous abortion, n (%)	1 (6.3%)	1 (9.1%)	2 (7.4%)	—
Negative pregnancy test, n (%)	11 (68.8%)	9 (81.8%)	20 (74.1%)	—
**Live birth rate (LBR), n (%)**	**4 (25.0%)**	**1 (9.1%)**	**5 (18.5%)**	**5/16 (31.2%)**

β-hCG, beta human chorionic gonadotropin; LBR, live birth rate; CPR, clinical pregnancy rate; ET, embryo transfer. LBR per transfer is defined as the number of live births divided by the number of transfer cycles performed. Cumulative LBR per patient is defined as the proportion of patients achieving at least one live birth across all transfer cycles. Bold values indicate the cumulative live birth rate (LBR) per patient, as distinct from the per-cycle LBR values reported in the same row.

With respect to endometrial thickness, no statistically significant difference was observed between patients achieving a positive β-hCG (mean 9.40 mm) and those with a negative result (mean 9.81 mm) at the first transfer cycle (p = 0.54). No patients with endometrial thickness <7 mm at transfer were included, as all met the ≥7 mm adequacy threshold before proceeding.

### 3.4 Subgroup analyses

**Table 5** presents the results of pre-specified subgroup analyses. By histological diagnosis, the LBR per transfer was 20.0% (4/20 ET) among EEC patients and 14.3% (1/7 ET) among AEH patients (p = 1.00, Fisher’s exact test). Within the EEC group, LBR per transfer was 18.8% (3/16 ET) for EEC endometrioid Grade 1, Stage IA without myometrial invasion and 25.0% (1/4 ET) for EEC endometrioid G2 stage IA without myometrial invasion, with no statistically significant difference (p = 1.00).

**Table 5:** Subgroup analysis of live birth rate by histological diagnosis, recurrence, and FST regimen.

Subgroup	Patients (n)	ET cycles (n)	Live births (n)	LBR/ET (%)	LBR/patient (%)
By histological diagnosis					
Endoemtrioid Endometrial carcinoma (EEC)	12	20	4	20.0%	33.3%
EEC Stage IA G1	9	16	3	18.8%	33.3%
EEC Stage IA G2	3	4	1	25.0%	33.3%
Atypical endometrial hyperplasia (AEH)	4	7	1	14.3%	25.0%
By disease recurrence					
No recurrence	7	11	3	27.3%	42.9%
Recurrence	9	16	2	12.5%	22.2%
By FST regimen					
Megestrol acetate + LNG-IUS	11	20	3	15.0%	27.3%
LNG-IUS alone	5	7	2	28.6%	40.0%

EEC, Endometrioid endometrial carcinoma; AEH, atypical endometrial hyperplasia; FIGO, International Federation of Gynecology and Obstetrics; FST, fertility-sparing treatment; LNG-IUS, levonorgestrel-releasing intrauterine system; LBR, live birth rate; ET, embryo transfer. p-values calculated using Fisher’s exact test. Due to the small sample size, all p-values should be interpreted with caution.

By disease recurrence status, patients without recurrence (n = 7) achieved a LBR per transfer of 27.3% (3/11 ET) and a cumulative LBR per patient of 42.9% (3/7), compared to 12.5% (2/16 ET) and 22.2% (2/9) respectively in patients with at least one recurrence episode (p = 0.38 for LBR/ET; p = 0.35 for LBR/patient). Although the differences did not reach statistical significance—likely attributable to the limited sample size—the magnitude of the observed effect (LBR/ET 27.3% vs. 12.5%; odds ratio 2.67) represents a clinically meaningful trend that warrants further investigation in larger prospective cohorts.

By FST regimen, patients treated with LNG-IUS alone had a numerically higher LBR per transfer (28.6%; 2/7 ET) compared to those receiving combined megestrol acetate plus LNG-IUS (15.0%; 3/20 ET; p = 0.57). This observation may reflect a less pronounced progestin-related endometrial toxicity with LNG-IUS monotherapy, though the small subgroup sizes preclude definitive conclusions.

## 4 Discussion

This retrospective cohort study presents, to our knowledge, the first series specifically characterizing blastocyst transfer outcomes following FST in women aged ≥40 years with EEC or AEH who subsequently underwent OD. The principal finding is that, despite consistently excellent embryological parameters, including 80.4% top-quality blastocysts and a 96.8% oocyte survival rate, and apparently adequate endometrial preparation, reproductive outcomes remained markedly impaired. Specifically, the cumulative LBR per embryo transfer was only 18.5% in patients with EEC, appears substantially lower than the 30–50% generally reported in standard OD programs involving women of comparable age without prior endometrial pathology (26). Consistently, SART registry data report live birth rates per transfer of approximately 35–45% in OD recipients aged 41–44 years, declining further in women aged ≥45 years, figures that compare unfavourably with the cumulative LBR of 31.2% per patient observed in our cohort despite optimal embryological parameters, further supporting the hypothesis of FST-related endometrial impairment superimposed on age-related uterine dysfunction (26).

The exclusive use of elective single eSET in all cycles further strengthens the interpretability of our findings by minimizing confounding related to multiple embryo transfer strategies.

Our findings extend and complement the observations reported by Friedlander et al. (27), who demonstrated significantly reduced reproductive outcomes in women with EEC or AEH undergoing embryo transfer after FST, even following transfer of euploid embryos. Their cohort comprised predominantly younger women using autologous oocytes, making the convergence of findings across these biologically distinct populations consistent with the interpretation that impaired endometrial receptivity, rather than embryo quality alone, represents the principal limiting factor in post-FST reproductive failure.

From an oncological perspective, these findings must be contextualized within the broader framework of FST as an alternative to hysterectomy, the standard of care for EC. Although hysterectomy confers definitive oncological control, it precludes any future pregnancy. Notably, even among patients who would otherwise be candidates for primary surgical treatment, a meaningful proportion who pursue FST ultimately achieve live birth, underscoring the clinical value of conservative management in carefully selected cases (23, 28).

Cheng et al. (28), in one of the largest retrospective cohorts evaluating pregnancy outcomes after FST in women with early-stage EEC or AEH (481 patients), reported overall pregnancy and live birth rates of 58.4% and 48.7%, respectively, with significantly higher reproductive success among patients undergoing ART compared with natural conception. However, ART pregnancies were associated with increased obstetric morbidity, and repeated hysteroscopic evaluations emerged as a probable independent risk factor for placenta accreta/increta, reinforcing the importance of limiting unnecessary surveillance procedures and underscoring concerns regarding cumulative endometrial injury from repeated intrauterine instrumentation (29–31).

The cumulative detrimental effect of repeated FST cycles and disease recurrence on endometrial function is corroborated by multiple independent lines of evidence. Chen et al. (32) reported that, in a cohort of 98 patients with recurrent EEC or AEH retreated with FST after an initial complete response, each additional round of conservative therapy was associated with progressively lower response rates and a reduced likelihood of successful pregnancy, with no pregnancies observed following a third round of treatment despite ART use. Chen et al. (26) demonstrated that, in progestin-resistant EEC/AEH patients, repeated FST cycles led to poor endometrial receptivity and lower subsequent pregnancy rates even after histological remission. Wang et al. (33) further confirmed that irregular menstruation and increased endometrial thickness post-pregnancy were linked to higher recurrence rates, highlighting the complex interplay between reproductive and oncological outcomes in this setting.

The timing of ART initiation following complete response represents a critical oncological and reproductive decision. Fan et al. (34) demonstrated that recurrence prior to pregnancy was highly detrimental to conception, with patients experiencing relapse being 80% less likely to conceive than those proceeding directly to ART after achieving complete response. Vaugon et al. (35) further demonstrated that IVF after conservative management did not increase recurrence risk and that pregnancy itself exerted a protective effect against recurrence, reinforcing the oncological rationale for early ART integration consistent with the recommendation by Cheng et al. (28) that ART should be initiated as early as possible after complete response.

Patients with recurrence in our cohort demonstrated lower LBR per transfer than those without, suggesting a cumulative detrimental effect of repeated FST cycles and prolonged progestin exposure on endometrial receptivity. Patients should be informed that each recurrence episode not only carries oncological risk but further compromises endometrial function and ART success, reinforcing the importance of timely definitive surgical treatment when fertility goals have been achieved or repeated recurrence occurs.

The pathophysiological basis of endometrial dysfunction after FST is likely multifactorial. High-dose progestin exposure has been shown to impair stromal decidualization, alter glandular architecture, and downregulate implantation-associated biomarkers including HOXA10, leukemia inhibitory factor (LIF), and integrins α-v and β-3 (36–38). In addition, repeated hysteroscopies and endometrial biopsies may induce chronic inflammation, intrauterine adhesions, and subclinical endometrial fibrosis, potentially compromising endometrial receptivity independently of oncological response. Moreover, obesity, insulin resistance, metabolic syndrome, and PCOS have all been independently associated with poorer FST response rates and longer time to complete remission and may further reduce reproductive success even when histological remission is achieved (39–44).

Although all patients achieved endometrial thickness ≥7 mm before embryo transfer, implantation and live birth rates remained suboptimal. This dissociation between morphological and functional endometrial assessment suggests that conventional ultrasound-based parameters may inadequately reflect true receptivity in women previously treated with FST. Fan et al. (34) confirmed that relapse before pregnancy exerted a strong independent negative effect on conception regardless of endometrial thickness. These findings highlight the urgent need for advanced functional tools, including endometrial receptivity arrays, proteomic profiling, and molecular biomarkers, to guide embryo transfer timing in this ontologically complex population.

The reproductive outcomes observed in our cohort compare unfavourably not only with standard OD programs but also with studies involving younger women with EEC or AEH undergoing IVF with autologous oocytes. Multiple reports describe live birth rates of 40–70% in younger women post-FST when ART is initiated promptly (45–51), substantially higher than those observed in our cohort, underscoring the compounding effect of advanced maternal age on an already compromised endometrial environment.

It is important to acknowledge that advanced maternal age independently compromises endometrial receptivity through well-documented molecular, cellular, and epigenetic mechanisms, including impaired stromal decidualization, accumulation of senescent and multiciliated endometrial epithelial cells, and downregulation of implantation-related biomarkers (11, 12, 15). In our cohort, with a median age of 45.5 years at first transfer, the contribution of age-related uterine dysfunction to the observed reproductive outcomes cannot be fully disentangled from the effects of FST itself. This represents an inherent limitation of the study population and further underscores the clinical complexity of this subgroup.

From a clinical and oncological standpoint, women with EEC or AEH pursuing FST, particularly those aged ≥40 years or with recurrent disease, must receive individualized counselling acknowledging the possibility of substantially reduced ART success despite optimal embryo quality, while recognizing that a clinically meaningful proportion do achieve live birth. Close interdisciplinary collaboration between reproductive endocrinologists and gynaecologic oncologists is essential to facilitate prompt ART transition after the first confirmed complete response, minimizing delays and repeated endometrial procedures. Novikova et al. (47) demonstrated that live births and LNG-IUD maintenance therapy improved disease-free survival after FST, suggesting that integrating reproductive and oncological goals is not only feasible but potentially synergistic.

The principal limitations of the present study include its retrospective design, limited sample size, and single-center setting, all of which reflect the inherent rarity of this clinical condition and restrict the generalizability of our findings. Importantly, the absence of an internal control group of age-matched OD recipients without prior endometrial pathology represents a significant methodological constraint. Although comparisons with published benchmarks for standard OD programs are informative, they cannot substitute for a matched internal comparator and preclude any causal attribution of impaired reproductive outcomes to FST-related endometrial dysfunction. Accordingly, the present study should be interpreted strictly as a descriptive, hypothesis-generating case series, and its findings as preliminary observations consistent with, rather than confirmatory of, the hypothesis of persistent endometrial dysfunction after FST. Heterogeneity in FST regimens and adjunctive therapies may have introduced additional variability. Furthermore, the absence of advanced endometrial receptivity assessments, including endometrial receptivity arrays or proteomic profiling, limits mechanistic interpretation. Prospective controlled studies incorporating matched OD recipients without prior endometrial pathology and advanced functional endometrial assessment tools are warranted to confirm and extend these preliminary observations. Nevertheless, the consistency between our findings and recent large-scale evidence, including Cheng et al. (28) and the growing body of molecular data, reinforces the clinical importance of recognizing persistent endometrial dysfunction as a potential cumulative consequence of FST, directly relevant to the risk-benefit counselling preceding any decision to defer definitive surgical treatment in women with EEC or AEH.

## Data Availability

The raw data supporting the conclusions of this article will be made available by the authors, without undue reservation.
